# Study on the Mechanism of Chemical–Mechanical Synergistic Removal of SiC Surfaces Based on Electrochemical Friction Wear of Grinding Wheel Pairs

**DOI:** 10.3390/mi17030307

**Published:** 2026-02-28

**Authors:** Lijie Wu, Zhijun Chen, Yangting Ou, Jiawen Yao, Hang Zhang, Qiusheng Yan, Jisheng Pan

**Affiliations:** 1 School of Electromechanical Engineering, Guangdong University of Technology, Guangzhou 510006, China; wulj115086@hanslaser.com (L.W.); chenzhijun@dlut.edu.cn (Z.C.); 2112301388@mail2.gdut.edu.cn (Y.O.); 2112301067@mail2.gdut.edu.cn (J.Y.); 18898846739@163.com (H.Z.); qsyan@gdut.edu.cn (Q.Y.); 2 Guangdong Han’s Semiconductor Equipment Technology Co., Ltd., Guangzhou 511458, China; 3 State Key Laboratory of High-Performance Precision Manufacturing, Dalian University of Technology, Dalian 116024, China

**Keywords:** single-crystal SiC, laser-exfoliated surface, friction and wear, electrochemical grinding, surface quality

## Abstract

With the advancement of SiC wafers toward 12 inches and innovations in laser cutting technology, new demands have emerged for SiC grinding techniques—namely, high efficiency, low loss, and low wear ratio. This paper investigates electrochemical-assisted grinding of SiC using a grinding wheel–SiC pair model system, examining the effects of electrolyte type, concentration, voltage, load, and rotational speed on wear behavior. Experimental results reveal that NaCl is the most effective electrolyte among the six candidates tested. In the NaCl system, wear behavior is strongly influenced by the interplay between voltage and rotational speed. At a constant voltage of 3 V, increasing the rotational speed to 600 rpm produces a wear area of 1911.93 μm^2^, while at a higher voltage of 7 V with a lower speed of 200 rpm, the wear area reaches 1301.96 μm^2^, indicating that optimal material removal requires synergistic matching of electrical and mechanical parameters. At 2 wt% NaCl, a sudden change in wear behavior occurs at 6–7 min, indicating a dynamic balance between oxide formation and mechanical removal. Rotational speed shows a turning point at 600 rpm, where the wear mechanism shifts significantly, marking the transition to a synergistically enhanced regime. EDS analysis confirms that Na_2_SO_4_ increases surface oxygen content by 54.4% compared to deionized water, demonstrating enhanced electrochemical oxidation. The optimal parameter window for synergistic removal is identified as 1–2 wt% NaCl, 5–7 V, 600 rpm, and 100–150 g. This study provides quantitative insights into the synergistic removal mechanism of SiC, offering a theoretical foundation for developing efficient, low-loss electrochemical grinding technologies.

## 1. Introduction

Silicon carbide (SiC), as a third-generation, wide-bandgap, high-temperature semiconductor material [1], possesses superior properties at room temperature, including a large bandgap width, high breakdown voltage, high thermal conductivity, high electron saturation drift velocity, strong radiation resistance, and excellent chemical stability [2]. It is the most mature and fastest-growing third-generation semiconductor to date, and is exceptionally well-suited for manufacturing high-frequency, high-speed, high-temperature, high-power, compact, and low-energy-consumption power electronic devices. It serves as a key core material and ideal substrate material in fields such as new energy vehicles, photovoltaic power generation, ultra-high voltage transmission, rail transit, and 5G communication base stations [3,4].

SiC-based chips hold immense application potential due to their ability to withstand more extreme operating conditions. With rapid advancements in electronic and material technologies, 8-inch single-crystal SiC wafers have achieved mass production, while 12-inch wafer fabrication technology has also been successfully developed and is poised to enter large-scale production. Compared to Si-based chips, SiC substrate chips offer superior performance but come with higher fabrication costs [5,6]. Wafer processing costs account for over half of the total expense, severely hindering the development of third-generation semiconductor chips [7]. SiC wafer fabrication typically involves wafer slicing, grinding/lapping, and polishing stages [8]. The slicing stage still primarily relies on diamond wire saw technology, which suffers from significant material loss, extensive residual damage, and low processing efficiency [9]. Emerging SiC laser cutting technology enables ultra-thin, ultra-low-damage separation of SiC wafers, significantly reducing material loss and damage while improving processing efficiency and wafer yield [10,11,12]. However, since laser focusing is required within the SiC ingot, the SiC surface must achieve a mirror-like finish with minimal subsurface damage to minimize the impact on laser energy. Due to the surface roughness, high material hardness, and chemical stability of SiC, a two-step process of coarse grinding followed by fine grinding is currently widely used for machining. However, this approach is inefficient, causes severe grinding wheel wear, and results in high processing costs. To address this, many researchers have explored methods to enhance grinding efficiency and quality while reducing wheel loss by modifying grinding wheel structures, improving grinding wheel performance [13,14], or adopting alternative grinding techniques [15,16,17].

Ding et al. [16] proposed a brazed diamond grinding wheel with a defined grain distribution. The results have shown that, under similar operating conditions, the brazed grinding wheel produced a smaller grinding force, force ratio, smoother ground surface, and lower surface roughness compared to an electroplated grinding wheel. Li [18] used laser processing to fabricate an end grinding wheel with orderly arranged abrasive particles, and the performance of the fabricated grinding wheel was compared with that of a conventional grinding wheel. The resulting grinding wheel showed enhanced effectiveness in comparison with the conventional grinding wheel in terms of reduced grinding force. Under the tested grinding conditions, the roughness of the ground surface was reduced by up to 47% compared to that of conventional grinding wheels. Tsai [19] presented a diamond lapping wheel for SiC to improve the material removal rate. The experimental results showed that the rate of material removal using the ceramic wheel binder was higher than that using the resin-bound wheels. These studies demonstrate that optimizing grinding wheel structure and binder composition can lead to measurable improvements in machine performance. Dai et al. [20] investigated the scratch morphology characteristics and formation mechanisms, revealing that fractures were more pronounced on the scratch sides created by conventional grinding (CG) and ultrasonic vibration-assisted grinding (UVAG), with poor morphology at the scratch bottom. Wu et al. [21] explored ductile grinding of SiC at a relatively higher material removal rate. Ductile grinding of SiC can be achieved through a combination of increasing the wheel speed and controlling the grinding depth. Moreover, the critical chip thickness for ductile grinding of SiC can be greatly improved under a higher grinding speed compared to conventional speed grinding. Despite these advancements in wheel design and process optimization, purely mechanical methods often encounter inherent limitations in simultaneously achieving high material removal rates, superior surface quality, and reduced tool wear when machining hard and brittle materials like SiC [22]. To overcome these challenges, external field-assisted grinding techniques have been increasingly explored. Chen et al. [23] proposed electrochemical jet-assisted grinding (EJAG) to enhance both the grinding efficiency and surface integrity of SiC. The grinding results show that with the assistance of EJAG, both the machining rate and wear resistance of the wheel can be improved (Sa = 2.18 nm). Cai et al. [24] utilized the method of molecular dynamics to explore the surface/subsurface damage mechanism of laser-assisted single-grain scratching of 4H-SiC crystals. The results demonstrate that as the laser’s intensity rises, the scratching force and stress decrease, and the depth of subsurface damage diminishes. These studies demonstrate that the use of external field-assisted SiC surface oxidation or softening followed by mechanical removal can significantly reduce grinding wheel wear, extend grinding wheel life, and achieve good machining quality while enabling more efficient material removal.

The above studies indicate that modifying grinding wheel structure and process parameters can improve SiC machining quality and reduce wheel wear, although the effects are not significant. Assisting the surface oxidation of SiC with an external field, followed by mechanical removal, can substantially reduce wheel wear, extend wheel life, and achieve superior machining quality [25,26]. However, achieving an optimal balance between the chemical reaction rate and the high material removal rate during grinding remains a key technical challenge [27], especially in electrochemically assisted grinding. If the electrochemical reaction is too violent, uneven electrochemical dissolution is triggered, which may lead to the formation of pits on the SiC surface or the bond interface, or preferential corrosion along the grain boundaries, destroying the integrity and uniformity of the surface [28,29]. At the same time, the electrochemical effect not only affects the workpiece, but also acts on the grinding wheel. If the voltage is too high, the grinding wheel will also undergo severe electrochemical dissolution or corrosion [30], causing the abrasive grains to fall off prematurely and shortening the life of the grinding wheel [31]. On the contrary, if the reaction is too mild, the oxidation effect on the SiC surface is insufficient and the mechanical removal process still dominates, resulting in continued severe tool wear and potential subsurface damage [32,33]. In summary, achieving this balance is critical to fully realize the potential of electrochemical–mechanical synergistic grinding of silicon carbide, but the interactions and synergistic effects between key process parameters that control this balance, such as voltage, electrolyte concentration, and grinding speed, remain unclear.

To address this gap, the present study introduces a novel approach that integrates electrochemical and friction-wear technologies using a grinding wheel–SiC pair model system [34]. Unlike traditional electrochemical grinding studies that focus on material removal rate or surface quality respectively, this work systematically studies the synergistic mechanism that controls the balance between electrochemical reaction and mechanical removal. By employing a simplified model system, the individual effects of key parameters—voltage, electrolyte concentration, load, and rotational speed—on the interface could be analyzed, and tribological and electrochemical behavior were isolated and quantified. Through comprehensive analysis of friction coefficient curves and wear morphology, this study provides a quantitative assessment of how environmental media and process parameters affect SiC material removal efficiency under synergistic conditions. The research results lay a theoretical foundation for the development of high-efficiency and low-damage electrochemical grinding technology and provide practical guidance for parameter selection in industrial applications.

## 2. Materials and Methods

### 2.1. Experimental Materials

**Figure 1:**
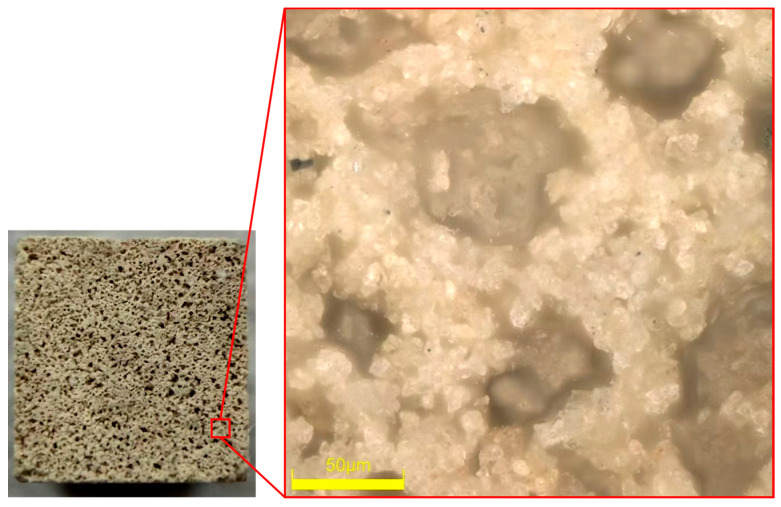
End-face morphology of grinding wheel blocks in a grinding pair.

Experimental chemical reagents include electrolyte types NaCl (AR, Aladdin, Shanghai, China), Na_2_S_2_O_8_ (AR, Aladdin), Na_3_PO_4_ (AR, Aladdin), and Na_2_SO_4_ (AR, Aladdin), and silica sol abrasive (SiO_2_, 100/150 nm, 40 wt%, ShanDong Peak-tech New Materials Co., Ltd., Linyi, China). To meet the sample mounting requirements of the friction and wear testing apparatus, 4H-SiC was first laser cut into 10 mm × 10 mm specimens, followed by grinding the Si surface to a surface roughness of Sa ≈ 3 nm. The surface oxygen content of the initial SiC substrate was 1.3–1.4%. For the grinding pair, a 2000 grit ceramic-bonded grinding wheel block developed by Hangzhou Xinyanke Semiconductor Co., Ltd. (Hangzhou, China). was used to cut the required grinding wheel blocks for the experiment. This grinding wheel block exhibits excellent wear resistance and a stable microstructure, serving as the key material for simulating the interaction between the grinding wheel and workpiece under actual machining conditions in the experiment (morphology shown in Figure 1). Subsequently, the grinding wheel blocks were machined into long-pin specimens of specified dimensions using cutting equipment to meet the requirements of subsequent friction and wear experiments. This machining process strictly controlled dimensions to ensure the geometric shape and surface quality of the grinding wheel pins complied with experimental standards. The final grinding wheel pins will serve as the mating pair in experiments, undergoing friction and wear testing with workpieces in a simulated electrochemical anodizing environment to investigate their removal behavior and action mechanisms during composite machining processes.

### 2.2. Experimental Principle

Single-crystal SiC is a material characterized by high hardness, high brittleness, and high chemical stability. Conventional chemical–mechanical polishing methods struggle to effectively oxidize the surface material of SiC. The anodic oxidation method employs SiC as the anode during electrolysis. Under the influence of an external electric field, an electrochemical reaction occurs on its surface as shown in Equation (1), rapidly forming a low-hardness oxide layer. This layer is then efficiently removed through mechanical action.

(1) $$\mathrm{SiC}+8{\mathrm{OH}}^{-}+8h^{+}\to {\mathrm{SiO}}_{2}+4H_{2}O+{\mathrm{CO}}_{2}$$

### 2.3. Principles and Apparatus of Friction and Wear

**Figure 2:**
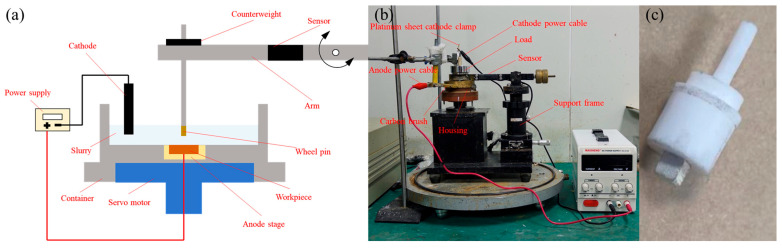
Electrochemical anodizing friction and wear test: (**a**) principle, (**b**) device, and (**c**) grinding wheel sample.

The experimental apparatus employed was the WTM-2E high-precision micro friction and wear tester (Lanzhou Zhongke Kaihua Science and Technology Development Co., Ltd., Lanzhou, China). The equipment primarily consists of a computer control console, main control box, and friction and wear test platform, enabling the characterization of material friction and wear properties under various environmental media. The principle of electrochemical friction and wear is illustrated in Figure 2. The electrochemical container is secured to the testing machine’s spindle along with the stage, driven by a motor for rotation. A ground SiC wafer is affixed to the center of the electrochemical container and connected to the positive terminal of a DC power supply as the anode. A platinum plate immersed in the slurry serves as the cathode.

A laser-shaped grinding wheel pin (uniform specifications to ensure experimental consistency) serves as the counter-grinding pair and is mounted on the support arm. The horizontal arm is adjusted via a balancing mechanism to ensure the grinding wheel pair contacts the SiC wafer under no-load conditions. The added load weights then represent the force exerted by the grinding ball onto the wafer. Tangential friction force is measured by a sensor and converted into a friction coefficient via the PC interface.

This experimental study focuses on investigating the influence of multiple key parameters on material removal performance, including grinding wheel pressure, relative motion speed, electrolyte type and concentration, and applied voltage magnitude. These parameters not only directly affect energy input and material response behavior during the friction and wear process but also significantly impact the formation rate and stability of the oxide layer. As a crucial intermediate product of electrochemical reactions, the oxide layer plays a vital role in the material removal process by “softening” the surface, reducing the friction coefficient, and regulating removal uniformity. Consequently, its formation is closely linked to material removal efficiency. The parameter ranges in Table 1 were determined through preliminary experiments: load (50–200 g) to cover typical contact pressures, rotational speed (200–600 rpm) based on equipment specifications, voltage (3–7 V) to avoid sparking while ensuring observable effects, and NaCl concentration (0.5–5 wt%) to maintain adequate conductivity without excessive corrosion. To comprehensively evaluate the effects of each parameter, multiple comparative conditions were established. High-precision measurement equipment was employed to quantitatively analyze key indicators such as the friction coefficient, wear volume, and surface morphology changes. Specific experimental conditions are detailed in Table 1.

## 3. Results

### 3.1. Influence of Different Electrolyte Systems on the Electrochemical Corrosion of SiC

**Figure 3:**
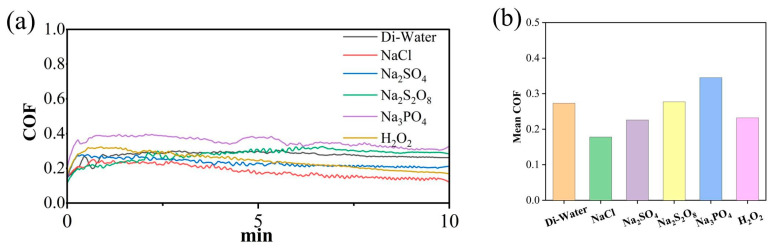
Friction and wear test results of SiC under different electrolytes: (**a**) COF curves (**b**) average COF.

As shown in Figure 3, the coefficient of friction (COF) curves of SiC wafers exhibit highly consistent overall trends across different electrolyte environments. Within the first 30 s of friction testing, the friction coefficient rises rapidly. This is primarily attributed to the initial adaptation process of the contact surfaces, including preliminary wear-in of surface roughness, rapid increase in actual contact area, and mutual interlocking effects between friction pairs. Subsequently, as the friction-wear process continues, the curve gradually stabilizes, indicating the system has entered a relatively stable friction-wear state where friction and wear behavior achieve dynamic equilibrium. Significant variations in the average coefficient of friction are observed across different electrolytes, closely related to their chemical reactivity at the friction interface, lubricating properties, corrosion behavior, and their ability to form or remove oxide films on material surfaces. The lower friction coefficient observed in NaCl solutions may be attributed to its strong electrolytic corrosion capability, which helps reduce surface adhesion effects and promotes uniform material removal. Conversely, the higher friction coefficient in strongly oxidizing electrolytes like Na_2_S_2_O_8_ may relate to their intense oxidation of surface oxide layers or poor lubricating properties, leading to enhanced cutting action or increased interface adhesion during friction.

**Figure 4:**
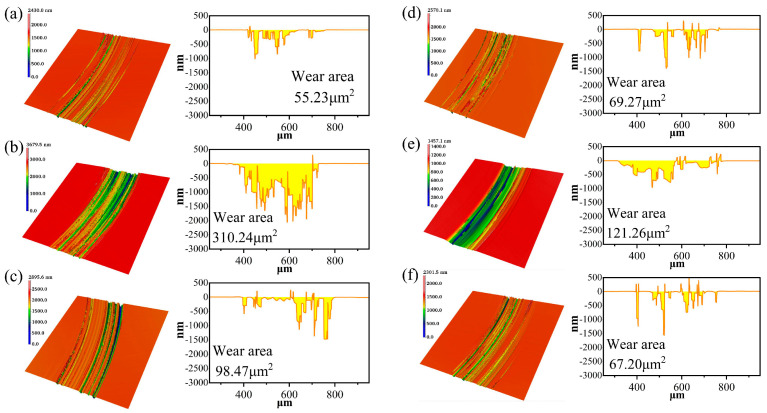
Wear morphology and wear zones of SiC under different electrolytes: (**a**) deionized water, (**b**) NaCl, (**c**) Na_2_SO_4_, (**d**) Na_2_S_2_O_8_, (**e**) Na_3_PO_4_, (**f**) H_2_O_2_.

**Figure 5:**
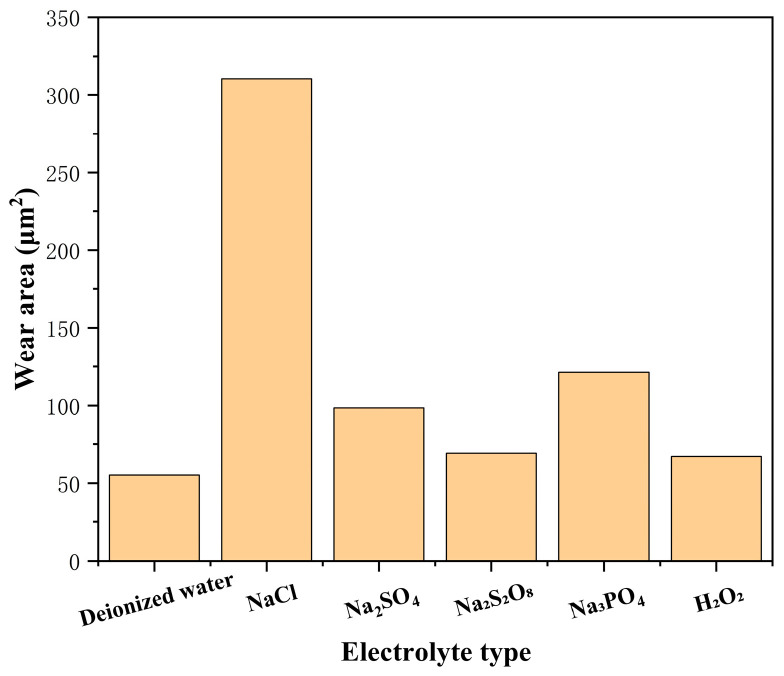
Statistical chart of wear area of SiC under different electrolyte types.

The wear morphology and wear area in different electrolytes are shown in Figure 4 and Figure 5. After friction and wear, distinct friction scratches appeared on the SiC wafer surface. Under different electrolyte conditions, the depth, width, and surface topography features of the scratches exhibited significant differences. In the NaCl electrolyte, the grinding wheel exerted a stronger cutting action on the SiC wafer, resulting in the largest area of wear. At this point, the SiC surface exhibited pronounced plow-furrow morphology caused by the grinding wheel pair, indicating the highest electrochemical oxidation rate of SiC material under this electrolyte environment, while the oxide layer was also more readily removed. In the deionized water test group, due to the poor conductivity of deionized water, electron exchange between the workpiece and the solution medium was reduced during electrochemical reactions, inhibiting the chemical corrosion efficiency of SiC, thus resulting in the smallest wear area. In Na_3_PO_4_ and Na_2_SO_4_ electrolytes, scratch depth decreased and surface wear was reduced. In the Na_2_S_2_O_8_ and 3% Fe-C + 5% H_2_O_2_ experimental groups, wear area further diminished with finer, shallower surface scratches. This suggests these electrolytes may have partially inhibited excessive material removal or promoted surface oxide film formation, thereby reducing direct mechanical wear.

### 3.2. Influence of Different Electrolyte Concentrations on the Electrochemical Corrosion of SiC

**Figure 6:**
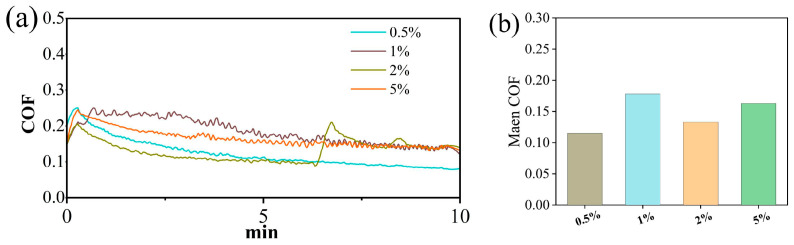
Friction and wear test results of SiC under different electrolyte concentrations: (**a**) COF curves (**b**) average COF.

**Figure 7:**
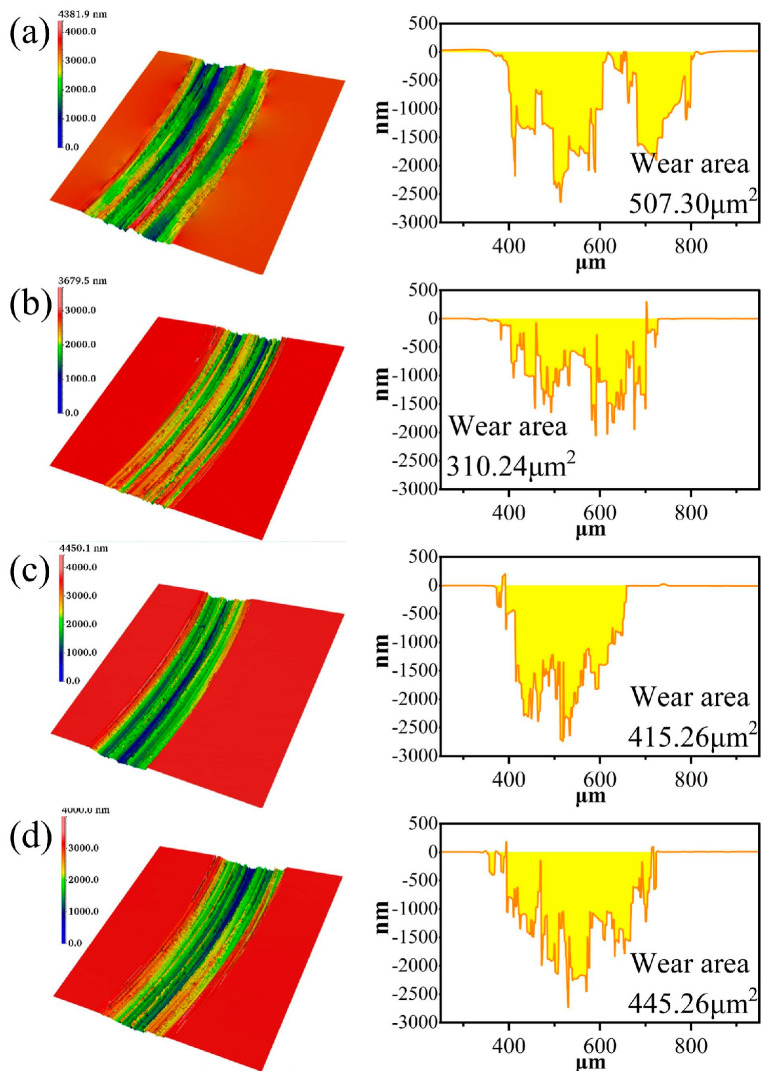
Wear morphology and wear zones of SiC under different electrolyte concentrations: (**a**) 0.5 wt% NaCl, (**b**) 1 wt% NaCl, (**c**) 2 wt% NaCl, (**d**) 5 wt% NaCl.

**Figure 8:**
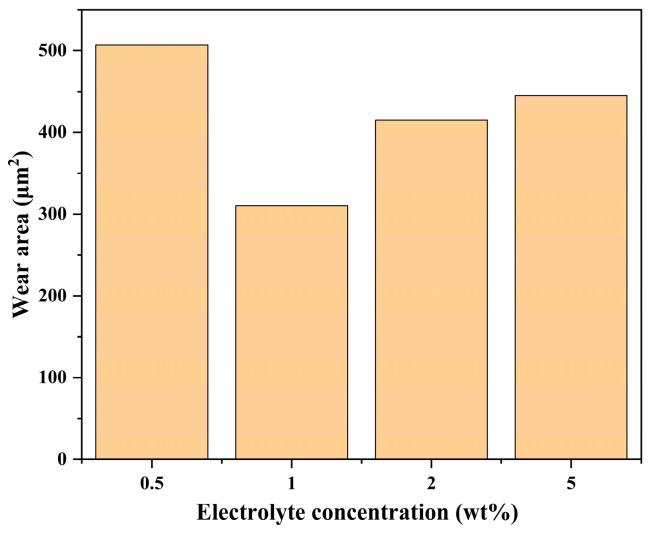
Statistical chart of wear area of SiC under different electrolyte concentrations.

After optimizing the NaCl electrolyte, the SiC friction coefficient curves and wear areas at different concentrations are shown in Figure 6, Figure 7 and Figure 8. The order of friction coefficient is: 1 wt% > 5 wt% > 2 wt% > 0.5 wt%. The electrolyte concentration affects the conductivity of the solution, which in turn affects the electron exchange rate between SiC and the solution, ultimately regulating the corrosion rate of SiC. At a concentration of 1%, the generation and removal of the oxide layer are in a critical equilibrium state, and the friction interface is the most unstable, so the friction coefficient is the highest. It is worth noting that at a concentration of 2 wt%, the friction coefficient curve shows a sudden jump at 6–7 min. This is due to the local peeling off of the accumulated oxide layer under mechanical action at 6–7 min, exposing the fresh SiC surface, resulting in an instantaneous increase in the friction coefficient. After peeling off, the surface is exposed to the electrolyte again, the oxide layer may be re-formed, and the friction coefficient becomes stable. This phenomenon is more typical at medium concentrations, because the oxide layer at low concentration (0.5 wt%) is thin, and the oxide layer at high concentration (5 wt%) is thick and stable; thus, 2 wt% is just in the transition zone where the oxide layer can break but is not completely covered. However, too high an electrolyte concentration (such as 5%) will cause the oxidation rate to be too fast, and the oxide layer will become thicker and difficult to remove. On the contrary, it will inhibit further corrosion of the SiC surface, thereby reducing the wear area. Therefore, at a low concentration of 0.5%, SiC has the deepest scratches, rough surface morphology, and obvious traces of material removal partially visible; while at a concentration of 2% or 5%, the scratched surface is relatively smooth, and the continuity and uniformity of the wear area have improved.

### 3.3. Influence of Different Voltages on the Electrochemical Corrosion of SiC

**Figure 9:**
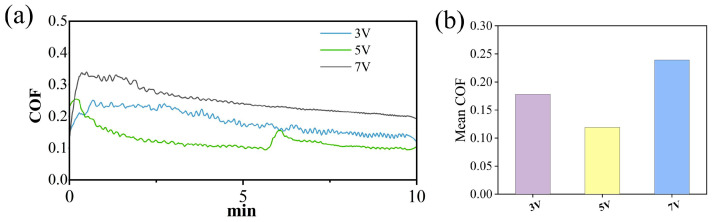
Friction and wear test results of SiC under different voltages: (**a**) COF curves (**b**) average COF.

**Figure 10:**
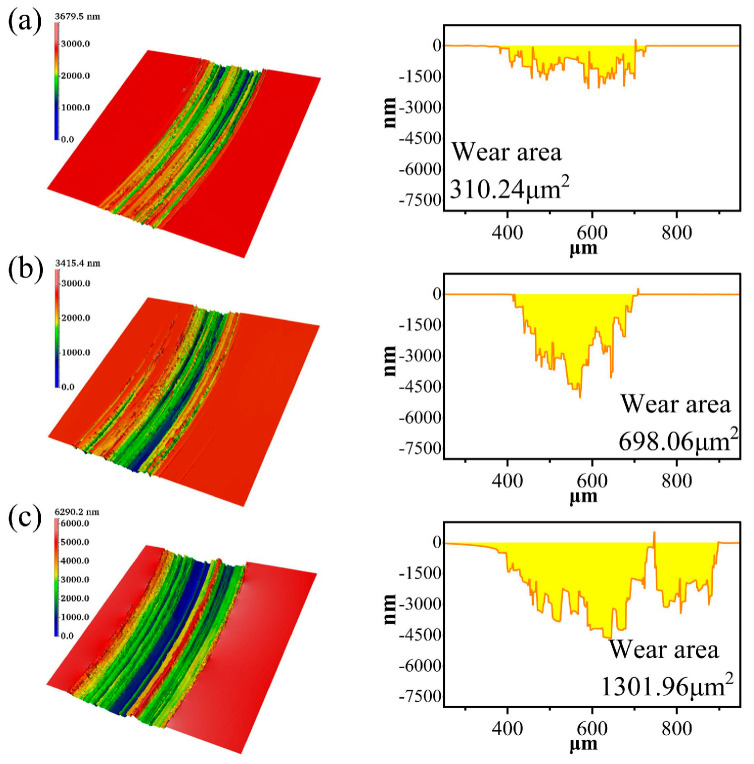
Wear morphology and wear areas of SiC under different voltages: (**a**) 3V, (**b**) 5V (**c**) 7V.

**Figure 11:**
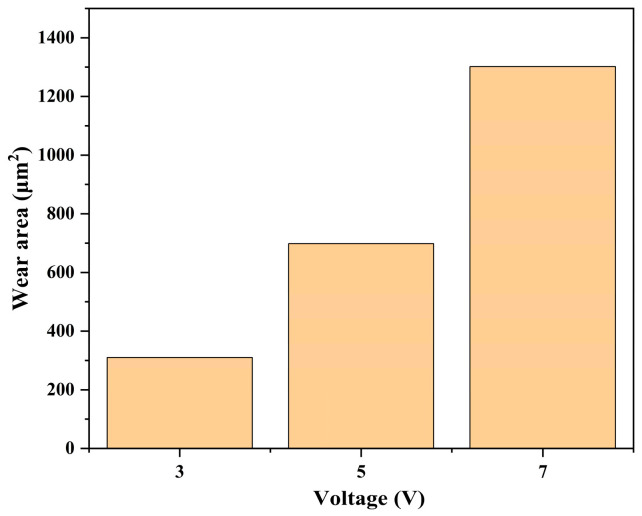
Statistical chart of wear area of SiC under different voltages.

The friction coefficient curves and wear areas of SiC under different voltages are shown in Figure 9, Figure 10 and Figure 11. The average friction coefficient was lowest at 5 V, followed by 3 V, with the highest friction coefficient observed at 7 V. When the voltage was set to 7 V, the material removal rate of the SiC wafer reached its maximum value of 1301.96 μm^2^, significantly higher than the removal effects under other voltage conditions. This indicates that at this voltage, the synergistic effect between electrochemical reactions and mechanical friction is more pronounced, enhancing material removal efficiency and improving process stability. This trend suggests that the interfacial friction between the grinding wheel and SiC material is significantly influenced by the applied voltage. As the voltage increases, the rate of electron transfer from the interior of the SiC to the anode accelerates, thereby increasing the corrosion rate. It also demonstrates that voltage is a key factor influencing the rate of electrochemical reactions. It is worth noting that at 5 V, the friction coefficient curve shows a sudden jump at 6–7 min. This phenomenon is related to the local shedding behavior of the oxide layer under this voltage. At a voltage of 5 V, the electrochemical reaction rate is moderate, and the formation of the oxide layer is similar to the mechanical removal rate. The oxide layer gradually accumulates during the friction process and reaches a critical thickness in 6–7 min. It is partially peeled off under mechanical action, exposing the fresh SiC surface, resulting in an instantaneous increase in the friction coefficient. After peeling off, the surface is exposed to the electrolyte again, the oxide layer is re-formed, and the friction coefficient becomes stable. In contrast, under 3 V, the oxide layer is thin and generated slowly, so it is not prone to obvious peeling; under 7 V, the oxide layer is generated quickly and is thick and stable, or it is regenerated immediately after being quickly removed, and the interface is in a continuously high reaction state, so there is no similar sudden jump.

### 3.4. Influence of Different Loads on the Electrochemical Corrosion of SiC

**Figure 12:**
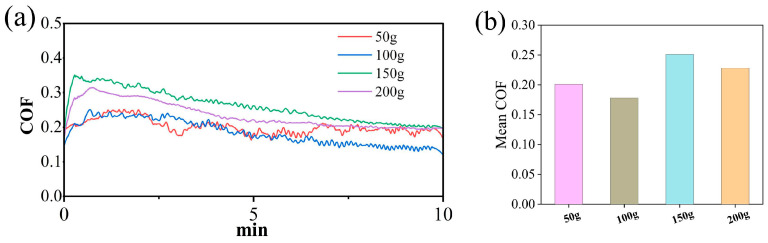
Friction and wear test results of SiC under different pressures: (**a**) COF curves (**b**) average COF.

**Figure 13:**
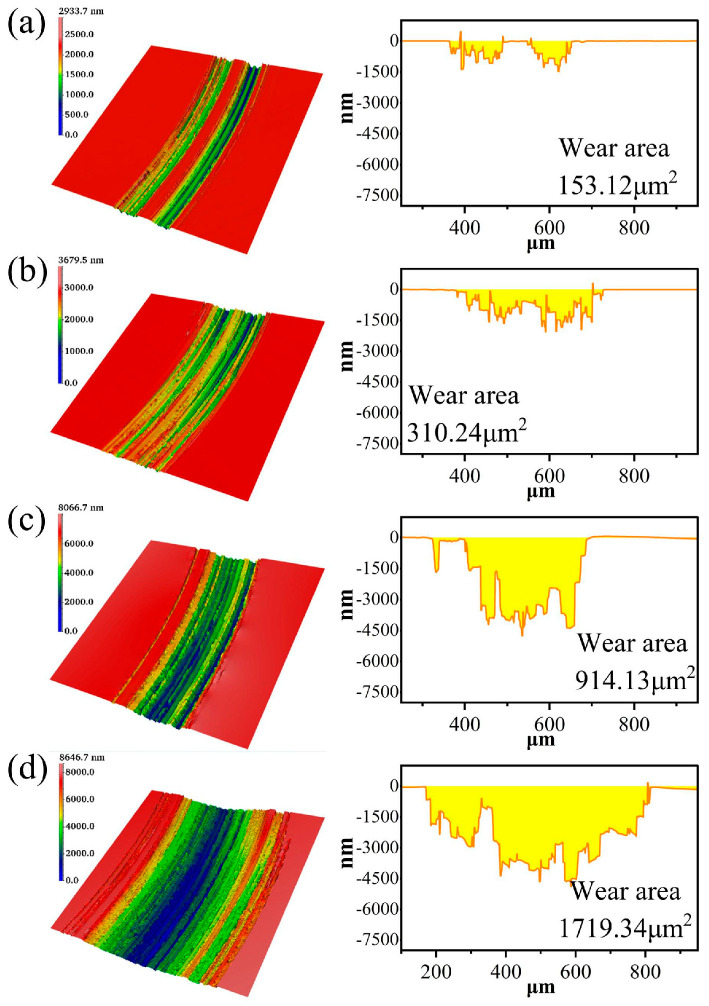
Wear morphology and wear zones of SiC under different pressures: (**a**) 50 g, (**b**) 100 g, (**c**) 150 g, (**d**) 200 g.

**Figure 14:**
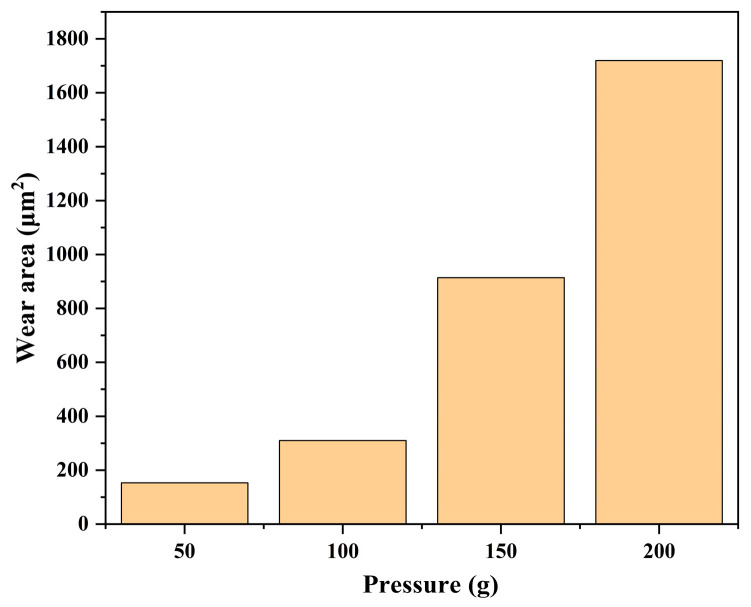
Statistical chart of wear area of SiC under different pressures.

The friction coefficient curves and wear areas of SiC under different loads are shown in Figure 12, Figure 13 and Figure 14. The average friction coefficient data indicates that the 100 g load exhibits the lowest average COF, followed by 50 g and 200 g, while the 150 g load yields the highest friction coefficient for SiC. Under lower pressure conditions, mechanical action intensity is weaker, resulting in slower material removal rates during friction and ineffective removal of the oxide layer. A stable, wear-resistant oxide layer readily forms at the contact interface, thereby reducing the friction coefficient. Consequently, scratch depth and width decrease sequentially, the surface becomes relatively flat, the continuity of the wear zone weakens, and the material removal process is moderated. Under higher pressure conditions, however, the abrasive particles in the grinding wheel pair penetrate deeper into the oxide layer, intensifying the cutting action. This results in a larger wear area and more severe surface damage. Under these conditions, oxide layer removal occurs rapidly. The grinding wheel pair comes into direct contact with the SiC substrate material, resulting in severe wear and a high coefficient of friction.

### 3.5. Influence of Different Rotational Speeds on the Electrochemical Corrosion of SiC

**Figure 15:**
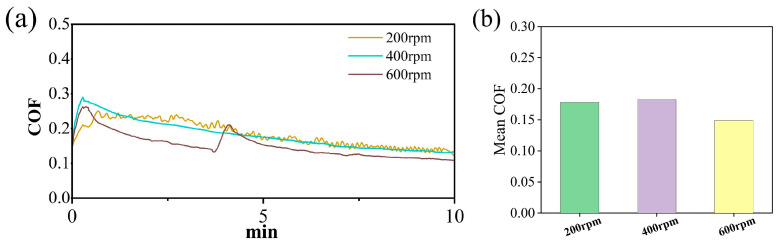
Friction and wear test results of SiC under different rotational speeds: (**a**) COF curves (**b**) average COF.

**Figure 16:**
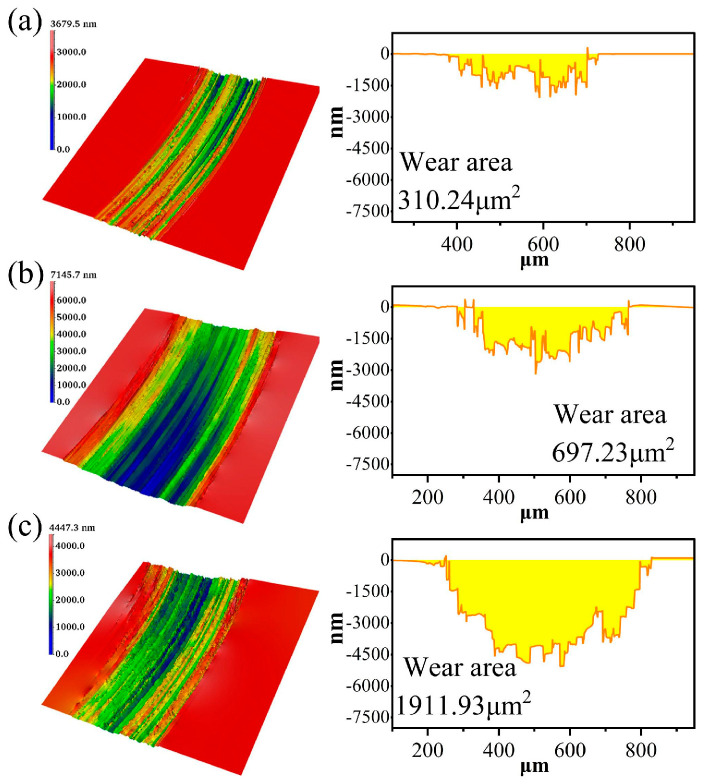
Wear morphology and wear areas of SiC under different rotational speeds: (**a**) 200 rpm, (**b**) 400 rpm, (**c**) 600 rpm.

**Figure 17:**
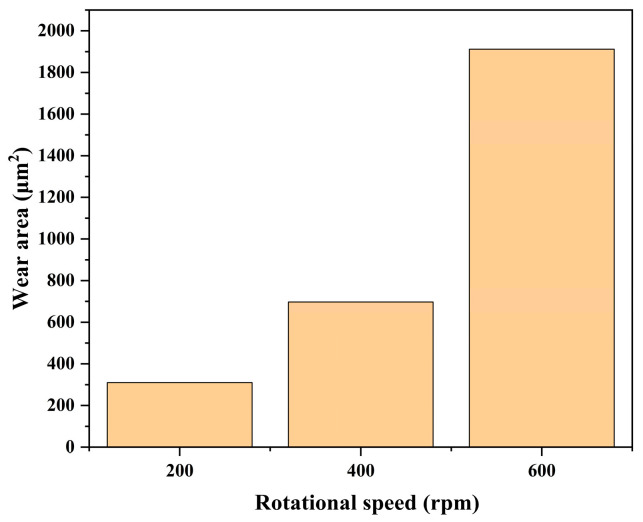
Statistical chart of wear area of SiC under different rotational speeds.

The friction coefficient curves and wear areas of SiC under different rotational speeds are shown in Figure 15, Figure 16 and Figure 17.

The friction coefficient was lowest at 600 rpm, followed by the 200 rpm experimental group, while the highest friction coefficient was observed at 400 rpm. These variations in friction coefficient may be closely related to thermal effects during friction, lubrication conditions, material removal mechanisms, and contact behavior between the grinding wheel and wafer. At 600 rpm, the friction coefficient curve shows a sudden jump at 4–5 min, which may be similar to the voltage and electrolyte concentration parameters mentioned above. There is a phenomenon that the oxide layer partially falls off under this condition, and, after peeling off, the oxide layer is re-formed, and the friction coefficient becomes stable. At the higher rotational speed of 600 rpm, the scratches were deepest and widest. At higher rotational speeds, the friction process may involve elevated surface temperatures and stronger mechanical impact forces, thereby accelerating material erosion and surface damage. The SiC surface exhibits significant damage, with pronounced plowing marks and localized material buildup, indicating intense cutting action between the grinding wheel and wafer at this speed. This results in a higher material removal rate during friction, leading to a larger wear area and more severe surface disruption. As the rotational speed decreased to 400 rpm and 200 rpm, the friction process became smoother. The interaction between the grinding wheel and the wafer tended to be milder, and the degree of wear correspondingly decreased. The scratches on the SiC surface gradually became shallower and narrower, the surface became relatively flat, the continuity of the wear area weakened, and the material removal process tended to stabilize. This may be related to the weakening of contact stress and thermal effects at the friction interface under lower rotational speeds.

## 4. Discussion

**Figure 18:**
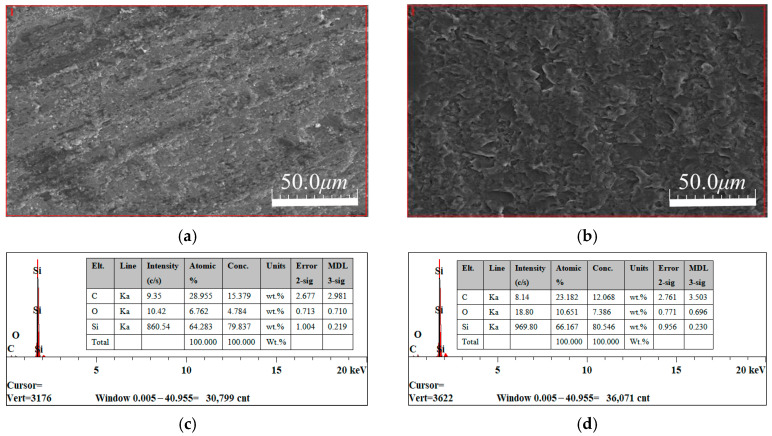
SEM image and EDS element distribution map at the wear track. (**a**) Wear trace images under deionized water. (**b**) Wear trace images under Na_2_SO_4_. (**c**) EDS elemental distribution map under deionized water. (**d**) EDS elemental distribution map under Na_2_SO_4_.

Figure 18 shows the scanning electron microscope (SEM) images and energy-dispersive X-ray spectroscopy (EDS) analysis of wear tracks on SiC when deionized water and Na_2_SO_4_ were used as electrolytes. Figure 18a reveals that under deionized water, the wear track exhibits a rough, uneven plow-furrow morphology with locally visible deep scratches and material spalling pits. This indicates that wear is primarily due to mechanical plowing, with material removal dependent on the mechanical action of dual surfaces or abrasive particles. In contrast, Figure 18b shows that under Na_2_SO_4_ electrolyte, the wear track surface is more uniformly smooth. The wear marks are shallower but cover a wider area, indicating that the wear mechanism shifts from purely mechanical plowing to a synergistic corrosion–wear interaction. Electrochemical corrosion participates in the weakening and removal of the material.

Comparing Figure 18c,d, it is evident that the Na_2_SO_4_ group exhibits a 54.4% increase in O content compared to the deionized water group. This indicates that the presence of the electrolyte induces more intense electrochemical oxidation reactions on the SiC surface, generating oxidation products such as SiO_2_ (as shown in Equation (1)). SiO_2_ exhibits significantly lower hardness than SiC, making it prone to microcrack initiation and spalling under friction, thereby accelerating material wear. The decrease in carbon content in the Na_2_SO_4_ group stems from the escape of carbon elements such as CO/CO_2_ during oxidation reactions, or the preferential dissolution and removal of surface carbon-based impurities (such as residual binders) due to electrochemical corrosion.

**Figure 19:**
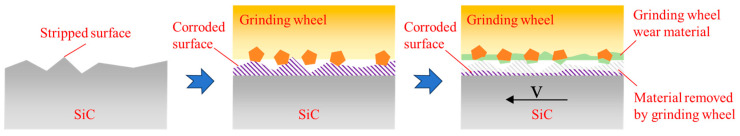
Mechanism of electrochemical–mechanical synergistic removal of single-crystal SiC in a grinding wheel–SiC pairing system.

Evidently, electrochemical corrosion represents an environmentally friendly chemical oxidation method capable of achieving efficient SiC oxidation. During electrochemical-assisted grinding, the subsurface damage layer remaining on the SiC surface from the cutting stage is efficiently oxidized under the combined action of the electric field and electrolyte, yielding a deep oxide layer. Subsequently, mechanical action from the grinding wheel efficiently removes this oxide layer, exposing fresh substrate material. This cyclic process of “oxidation–removal–reoxidation” enables electrochemical–mechanical grinding to maintain material removal efficiency while effectively controlling surface damage. This facilitates high-precision, high-efficiency machining of hard and brittle materials like single-crystal SiC, as shown in Figure 19. Furthermore, the relatively low hardness of the oxide layer significantly reduces grinding wheel wear while enhancing material removal rates, thereby extending the service life of the grinding wheel.

## 5. Conclusions

This study systematically analyzed the influence of electrolyte type, electrolyte concentration, voltage, pressure, and rotational speed on the electrochemical corrosion behavior of grinding wheel pairs towards monocrystalline SiC, using indicators such as the coefficient of friction (COF) curve and wear area. By measuring the COF variation curve during the friction process and the morphology and wear area of the sample surface after wear, the effects of various experimental parameters on the surface corrosion behavior and material removal process of monocrystalline SiC were evaluated. The main conclusions are as follows:

(1) Electrolyte type significantly influences the electrochemical corrosion behavior of single-crystal SiC, with the NaCl system exhibiting the most favorable overall performance. Increasing solution concentration generally enhances solution conductivity, thereby accelerating electrochemical reaction rates. However, excessively high concentrations may exacerbate corrosion effects, ultimately impairing material removal efficiency.
(2) Voltage was identified as a key parameter affecting the synergistic electrochemical–mechanical removal process of SiC. In the experimental test range of 3–7 V, as the voltage increases, the friction coefficient shows a downward trend, which can be attributed to the electrochemical reaction promoting the formation of the surface oxide layer. At the highest voltage tested, 7 V, a significant increase in the wear area was observed, indicating an increase in local electrochemical corrosion in this range. However, since this study did not test voltages higher than 7 V, subsequent in-depth studies using higher voltage levels are needed to determine whether this trend is a true threshold effect or a continuous change outside the test range.
(3) Load and rotational speed determine the intensity of mechanical removal. Under electrochemical assistance, appropriate pressure enhances current transfer efficiency between electrodes, thereby improving surface reaction uniformity. Higher rotational speeds facilitate rapid electrolyte renewal, reducing localized concentration polarization. This improves electrochemical corrosion performance and increases material removal rates.

## Figures and Tables

**Table 1:** Friction and Wear Test Conditions.

Serial Number	Load (g)	Rotational Speed (rpm)	Voltage (V)	Electrolyte	Concentration (wt%)
1	100	200	3	Di-water, NaCl, Na_2_SO_4_, Na_2_S_2_O_8_, Na_3_PO_4_, 3 wt% Fe-C + 5 wt% H_2_O_2_	1
2	100	200	3	NaCl	0.5, 1, 2, 5
3	100	200	3, 5, 7	NaCl	1
4	50, 100, 150, 200	200	3	NaCl	1
5	100	200, 400, 600	3	NaCl	1

## Data Availability

The original contributions presented in this study are included in the article. Further inquiries can be directed to the corresponding author.
